# Acute renal injury in ANCA-associated glomerulonephritis: etiology related to acute tubulointerstitial injury versus glomerular injury

**DOI:** 10.1080/0886022X.2026.2632436

**Published:** 2026-03-15

**Authors:** Hong-Shan Chen, Xin-Yue Shi, Zhen-Jun Zhao, Hao-Miao Zhang, Zi-Yi Sun, Yu-Nan Li, Li-Yuan Xie, Yang Xue, Li Wei, You-Xia Liu, Peng-Cheng Xu, Jun-Ya Jia

**Affiliations:** a Department of Nephrology, Kidney Disease Medical Center, General Hospital, Tianjin Medical University, National Key Clinical Specialty, Tianjin Key Medical Discipline, Tianjin, China; b Department of Rheumatology and Immunology, The Affiliated Hospital of Inner Mongolia Medical University, Hohhot, Inner Mongolia, China

**Keywords:** Anti-neutrophil cytoplasmic antibody-associated glomerulonephritis, tubulointerstitial injury, acute kidney injury, IgA nephropathy

## Abstract

Acute kidney injury (AKI) is common in anti-neutrophil cytoplasmic antibody-associated glomerulonephritis (ANCA GN). Although ANCA GN has crescents in 90% of cases, the crescentic form occurs in only 20 to 30%, so it is challenging to attribute acute kidney injury (AKI) solely to the glomerular component. In contrast, IgA nephropathy (IgAN), while also frequently demonstrating crescent formation, is associated with a notably lower incidence and severity of AKI compared to ANCA GN. This discrepancy suggests that acute tubulointerstitial injury may constitute a more prominent—and previously underrecognized—contributor to AKI in ANCA GN. This study aimed to compare tubulointerstitial injury between ANCA GN and IgAN after matching for glomerular injury, and to assess its impact on AKI in ANCA GN. Propensity score matching were used to compare 48 ANCA GN patients and 48 IgAN patients, matched for age, sex, and glomerular injury parameters. We compared the disparities in tubulointerstitial injury in the renal pathology of ANCA GN and IgAN and analyzed the influence of tubulointerstitial injury on AKI in ANCA GN. ANCA GN had more severe acute tubulointerstitial injury, alongside higher serum creatinine (*p* = 0.048) and AKI incidence (31.3% vs 4.2%, *p* < 0.001) than IgAN. In patients with ANCA GN, whether the influence of glomerular injury is corrected, patients with interstitial cell infiltration > 25% exhibit higher levels of serum creatinine. Acute tubulointerstitial injury is a major contributor to AKI in ANCA GN, independent of glomerular injury, highlighting its clinical significance in the acute disease phase.

## Introduction

Anti-neutrophil cytoplasmic antibody (ANCA) associated vasculitis (AAV) is a small-vessel vasculitis with multi-organ involvement. ANCA-associated glomerulonephritis (ANCA GN) is characterized by the formation of crescent lesions and/or fibrinoid necrosis with pauci-immune complexes [1]. Up to over 50% of ANCA GN patients present as acute kidney disease (AKI) at the onset of disease [2–4]. Although crescent formation can be detected in over 90% of renal biopsy specimens in ANCA GN, only 20–30% fulfill the criteria for crescentic nephritis (the proportion of glomerular crescent formation is ≥50%) [5]. Thus, it appears challenging to attribute the cause of AKI in ANCA GN exclusively to ‘severe glomerular injury’. At present, the pathological classification criteria for ANCA GN does not incorporate parameters of interstitial tubular injury. Nevertheless, several reports have confirmed that some patients exhibit remarkably acute renal interstitial tubular injury that is disproportionately pronounced in comparison to the extent of glomerular injury [6,7]. However, the impact of renal interstitial tubular injury on AKI in ANCA GN patients remains poorly understood.

In order to evaluate the contribution of tubulointerstitial injury to the AKI in patients with AAV, we conducted a comparative analysis between ANCA GN and another type of glomerulonephritis, immunoglobulin A nephropathy (IgAN). Although the pathological feature of IgAN is IgA deposition and cellular proliferation in the glomerular mesangial region, up to 30-50% of patients present with crescent formation at the time of receiving kidney biopsy [8,9]. Some cases even meet the criteria of crescentic nephritis [10]. However, both the incidence and severity of AKI in IgAN are significantly lower than those in ANCA GN [11]. We hypothesize that this may be associated with the relatively less severe acute renal tubulointerstitial injury in IgAN.

Therefore, this study aimed to compare the severity of acute tubulointerstitial injury between ANCA GN and IgAN with crescents (and/or fibrinoid necrosis) after matching for key glomerular injury parameters, and to evaluate the impact of such injury on the development of AKI in ANCA GN.

## Materials and methods

### Patients

**Figure 1.**
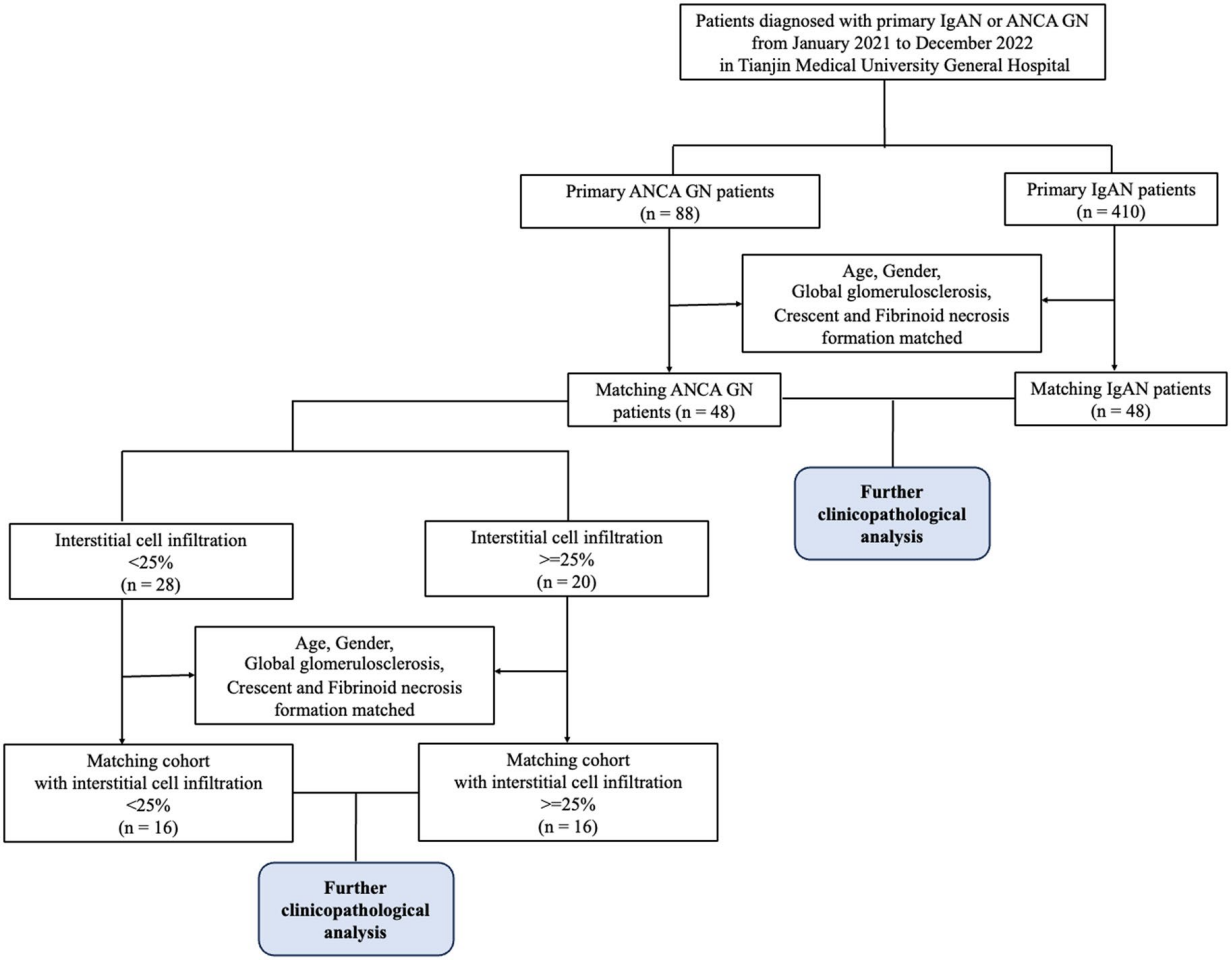
Flow diagram of the study population.

This was a retrospective cohort study. Patients diagnosed with either primary ANCA GN or IgAN at Tianjin Medical University General Hospital from January 2021 to December 2022 was selected for further analysis. Patients with ANCA GN combined with systemic lupus erythematosus (SLE) and other connective tissue diseases or complicated with any other primary and secondary glomerular disease were excluded. Patients with IgAN overlapped with Henoch–Schonlein purpura, SLE, liver disease or complicated with ANCA GN as well as positive ANCA serology were also excluded. Finally, 88 patients with biopsy-proven ANCA GN and 410 patients diagnosed as primary IgAN with detailed baseline characteristics and more than 6 glomeruli in biopsy specimens were enrolled. Using propensity score matching (PSM) method, we selected 48 ANCA GN and 48 IgAN patients who were age, sex, the proportion of global glomerulosclerosis, cellular crescent formation and fibrinoid necrosis formation in renal biopsies matched for further baseline and follow-up data analysis. Firstly, we compared the differences in renal interstitial injury between ANCA GN and IgAN under the condition of the same degree of crescent formation. Then we conducted a further analysis to assess the impact of renal interstitial cell infiltration on renal function within 48 patients with ANCA GN. Among 48 ANCA GN patients, 28 patients had a percentage of interstitial cell infiltration area less than 25% and 20 patients had a percentage of interstitial cell infiltration area over 25%. We selected 16 age, sex and glomerular injury degree matched patients from each group through 1:1 propensity score matching (PSM) (Figure 1). This study was performed in adherence with the Declaration of Helsinki and with the approval of the Ethics Committee of Tianjin Medical University General Hospital (IRB2025-KY-270).

### Clinical data collection and definition

Baseline demographic characteristics and clinical data including age, sex, routine blood analysis, serum creatinine, serum albumin, serum D-dimer, C-reaction protein (CRP), erythrocyte sedimentation rate (ESR), C3, C4, routine urinary analysis, proteinuria and hematuria. The baseline level of urinary neutrophil gelatinase associated lipocalin (uNGAL), a well-established early biomarker of acute tubular injury [12], and urinary N-acetyl-β-D-glucosaminidase (uNAG), a sensitive indicator of proximal tubular damage reflecting lysosomal activity were also collected [13]. AKI was defined by KDIGO definition, serum creatinine increases by ≥ 0.3 mg/dL (≥ 26.5 μmol/L) within 48 h, or increase ≥ 1.5 times baseline known or presumed to have occurred within the previous 7 days; or urine output < 0.5 mL/kg per h for 6 h [14]. Vasculitis disease activity among ANCA GN patients was recorded using the Birmingham Vasculitis Activity Score (BVAS) 2003.

### Pathological data collection and definition

Renal tissue was procured via percutaneous needle biopsy. All specimens were processed according to standard protocols for examination by light microscopy, immunofluorescence microscopy, and electron microscopy. Tissue sections were stained with hematoxylin and eosin (HE), periodic acid–Schiff (PAS), periodic acid–Schiff methenamine silver (PASM), and Masson’s trichrome. Two independent renal pathologists, who were blinded to all clinical information, performed the histological assessment. Crescent fraction was defined as the fractions of glomeruli with cellular or fibrocellular crescents. Fibrinoid necrosis fraction was measured by the number of glomeruli with fibrinoid necrosis lesions in total glomeruli. Global glomerulosclerosis fraction was the percentage of glomeruli with global sclerosis. Bowman’s capsule rupture was defined as discontinuity of the basement membrane of Bowman’s capsule. Mesangial hypercellularity was assessed according to the Oxford Classification criteria and considered present if more than half of the glomeruli contained more than three mesangial cells in a mesangial area. Endocapillary hypercellularity was assessed according to the Oxford Classification criteria, defined as the presence of increased cellularity within glomerular capillary lumina—including endothelial cells, monocytes, and/or neutrophils—leading to visible luminal narrowing or occlusion in any non-sclerotic glomerulus. Each case was classified as present (1) or absent (0). Segmental glomerulosclerosis was defined as the appearance sclerosis or adhesion involving only a portion of the glomerular tuft, assessed exclusively in non-globally sclerotic glomeruli. Each case was classified as present (1) or absent (0). The degree of loss of tubular brush border, interstitial cell infiltration, tubular atrophy, and interstitial fibrosis was classified by the percentage of <25%, 25%–50% and >50%.

### Immunohistochemistry

Formalin fixed, paraffin-embedded kidney tissues were treated by appropriate predetermined antigen retrieval methods, heating in an autoclave containing EDTA solutions. Then, sections were treated in 3% hydrogen peroxide for 0 to 5 min at room temperature in the dark and were placed in 3% bovine serum albumin. The liquid was shaken off directly and then treated with mouse anti-CD68 monoclonal antibody (ZSGB Bio, PRC) for 2 h at room temperature. After washing, the sections were incubated with the horseradish peroxidase-conjugated secondary antibody for 30 min at room temperature. Subsequently, they were stained with diaminobenzidine and counterstained with Mayer’s hematoxylin. Finally, the sections underwent dehydration and clearing in alcohol and xylene. Pictures were taken with an optical microscope (Nikon, Japan).

### Treatment and outcome definition

Renin-angiotensin system inhibitor (RASI) treatment was defined as the use of an angiotensin-converting enzyme inhibitor and/or angiotensin receptor blocker after renal biopsy. Glucocorticoid treatment referred to the use of any type of systemic glucocorticoid (e.g. methylprednisolone, prednisone), irrespective of specific dosage or duration. Immunosuppressive agents treatment was defined as the use of immunosuppressive agents such as cyclophosphamide, mycophenolate mofetil, and cyclosporine regardless of dose. Rituximab treatment was initiated with an intravenous infusion of total 2 g. Patients who had a ≥50% reduction in eGFR, ESRD (eGFR < 15 mL/min/1.73 m^2^), maintained chronic dialysis over 6 months or received renal transplantation was defined as reaching renal endpoint.

### Statistical analyses

Categorical variables are described as frequencies and tested by the chi-squared test. Normality-distributed continuous variables are expressed as means along with SD, while nonparametric variables are described as medians with interquartile ranges (IQR). Student’s t-test or Mann–Whitney U-test was used to compare the statistical difference. Univariate and multivariate logistic regression models were employed to evaluate the impact of tubulointerstitial injury parameters on AKI and to identify potential risk factors for severe interstitial cell infiltration. A two-tailed *P*-value < 0.05 was thought statistically significant. SPSS (IBM, version 26.0) and R software (version 4.1.2) were used for statistic analysis. Graphical representations were generated using GraphPad Prism (version 9.0).

## Results

### After matching, ANCA GN had more severe tubulointerstitial injury in renal pathology than IgAN

As shown in Table 1, there were no significant differences in crescent fraction (0.14 [0.03, 0.24] vs 0.14 [0.07,0.22], *p* = 0.912), fibrinoid necrosis fraction (0.04 [0.00, 0.09] vs 0.00 [0.00, 0.05], *p* = 0.068), global glomerulosclerosis (0.00 [0.00, 0.12] vs 0.00 [0.00, 0.08], *p* = 0.299) and Bowman’s capsule rupture (10.4% vs 2.1%, *p* = 0.092) between ANCA GN and IgAN. At the meanwhile, IgAN patients showed more severe glomerular injury including mesangial hypercellularity (*p* < 0.001), endocapillary hypercellularity (*p* < 0.001), and segmental glomerulosclerosis (*p* < 0.001). However, the degree of interstitial tubular injury in ANCA GN patients was more severe than that in IgAN patients. The extent of loss of brush border and interstitial cell infiltration in patients with ANCA GN was greater than those in patients with IgAN (*p* = 0.006 and *p* < 0.001). There were no differences in tubular atrophy and interstitial fibrosis between the two groups (*p* = 0.669 and *p* = 0.898). Therefore, compared with IgAN, patients with ANCA GN experience more severe acute renal tubulointerstitial injury, while there are no disparities in chronic renal tubulointerstitial injury between ANCA GN and IgAN.

**Table 1.** Comparison of pathological characteristics between ANCA GN and IgAN.

Parameter	ANCA GN (*N* = 48)	IgAN (*N* = 48)	*p* value
Sex (M/F)	18/30	20/28	0.835
Age (years)	56.08 ± 16.55	55.31 ± 12.37	0.363
Glomeruli number, n	21.00 [14.00, 25.25]	13.00 [11.00, 18.25]	**<0.001**
Crescent fraction	0.14 [0.03, 0.24]	0.14 [0.07, 0.22]	0.912
Fibrinoid necrosis fraction	0.04 [0.00, 0.09]	0.00 [0.00, 0.05]	0.068
Global glomerulosclerosis fraction	0.00 [0.00, 0.12]	0.00 [0.00, 0.08]	0.299
Bowman’s capsule rupture, n (%)			0.092
0	43 (89.6)	47 (97.9)	
1	5 (10.4)	1 (2.1)	
Mesangial hypercellularity, n (%)			**<0.001**
<50%	28 (58.3)	1 (2.1)	
>50%	20 (41.7)	47 (97.9)	
Endocapillary hypercellularity, n (%)			**<0.001**
0	44 (91.7)	28 (58.3)	
1	4 (8.3)	20 (41.7)	
Segmental glomerulosclerosis, n (%)			**<0.001**
0	45 (93.8)	27 (56.2)	
1	3 (6.2)	21 (43.8)	
Loss of brush border, n (%)			**0.006**
0	6 (12.5)	16 (33.3)	
0-25%	34 (70.8)	31 (64.6)	
25-50%	8 (16.7)	1 (2.1)	
>50%	0 (0.0)	0 (0.0)	
Interstitial cell inflammation, n (%)			**<0.001**
0	0 (0.0)	0 (0.0)	
0-25%	28 (58.3)	47 (97.9)	
25%-50%	20 (41.7)	1 (2.1)	
>50%	0 (0.0)	0 (0.0)	
Tubular atrophy, n (%)			0.669
0	2 (4.2)	0 (0.0)	
0-25%	28 (58.3)	32 (66.7)	
25-50%	14 (29.2)	12 (25.0)	
>50%	4 (8.3)	4 (8.3)	
Interstitial fibrosis, n (%)			0.898
0	0 (0.0)	0 (0.0)	
0-25%	29 (60.4)	32 (66.7)	
25-50%	15 (31.2)	12 (25.0)	
>50%	4 (8.3)	4 (8.3)	

**Abbreviations** M/F: Male/Female.

### After matching, the clinical manifestations of kidney injury in ANCA GN are more severe than those in IgAN

As shown in Table 2, ANCA GN patients suffered more severe renal function decline than IgAN patients, presented as higher level of Scr (105.00 [74.75, 154.25] vs 92.00 [69.75, 118.25] umol/L, *p* = 0.048) and higher incidence of AKI (31.25% vs 4.17%, *p* < 0.001). Among these AKI patients, 2 of the ANCA GN suffered dialysis from onset while no IgAN patients need renal replace treatment. Compared with IgAN patients, AAGN demonstrated significantly elevated uNAG levels (22.60 [14.00, 33.40] vs 14.40 [8.80, 22.10] U/gcr, *p* = 0.006), with a numerical trend toward higher uNGAL concentrations (29.30 [15.76, 45.14] vs 25.17 [13.52, 38.08] ng/mL, *p* = 0.333) and reduced urinary specific gravity (1.01 ± 0.01 vs. 1.02 ± 0.01, *p* = 0.132). There is no significant difference in hematuria and proteinuria levels between ANCA GN patients and IgAN patients (*p* = 0.904 and *p* = 0.836).

**Table 2.** Comparison of the clinical indices of kidney injury between ANCA GN and IgAN.

Parameter	ANCA GN (*N* = 48)	IgAN (*N* = 48)	*p* value
Scr (μmol/L)	105.00 [74.75, 154.25]	92.00 [69.75, 118.25]	**0.048**
AKI, n (%)	15 (31.25)	2 (4.17)	**<0.001**
Dialysis from onset, n (%)	2 (13.34)	0 (0.00)	0.582
uSG	1.01 ± 0.01	1.02 ± 0.01	0.132
uRBC (/μL)	163.20 [23.30, 346.15]	126.80 [59.70, 257.15]	0.904
Proteinuria (g/24h)	1.09 [0.68, 2.00]	1.06 [0.61, 1.68]	0.836
uNAG (U/gcr)	22.60 [14.00, 33.40]	14.40 [8.80, 22.10]	**0.006**
uNGAL (ng/mL)	29.30 [15.76, 45.14]	25.17 [13.52, 38.08]	0.333
Treatment			
RASI Treatment, n (%)	5 (10.42%)	20 (41.67%)	**<0.001**
Glucocorticoid treatment, n (%)	38 (79.17%)	33 (68.75%)	0.245
Immunosuppressive agents treatment, n (%)	19 (39.58%)	24 (50.00%)	0.305

**Abbreviations** Scr: Serum creatinine; AKI: Acute kidney injury; uSG: Urinary specific gravity; uRBC: Urinary red blood cell; uNAG: Urinary N-acetyl-β-D-glucosaminidase; uNGAL: Urinary neutrophil gelatinase-associated lipocalin; RASI Renin-angiotensin system Inhibitor treatment.

Regarding treatment, a significantly higher proportion of IgAN patients received RASI therapy compared to ANCA GN patients (41.67% vs. 10.42%, *p* < 0.001). The use of glucocorticoids (68.75% vs. 79.17%, *p* = 0.245) and immunosuppressive agents (50.00% vs. 39.58%, *p* = 0.305) did not differ significantly between the two groups.

### Among all four parameters representing renal interstitial tubular injury, only the interstitial cell infiltration in ANCA GN is associated with AKI

**Figure 2.**
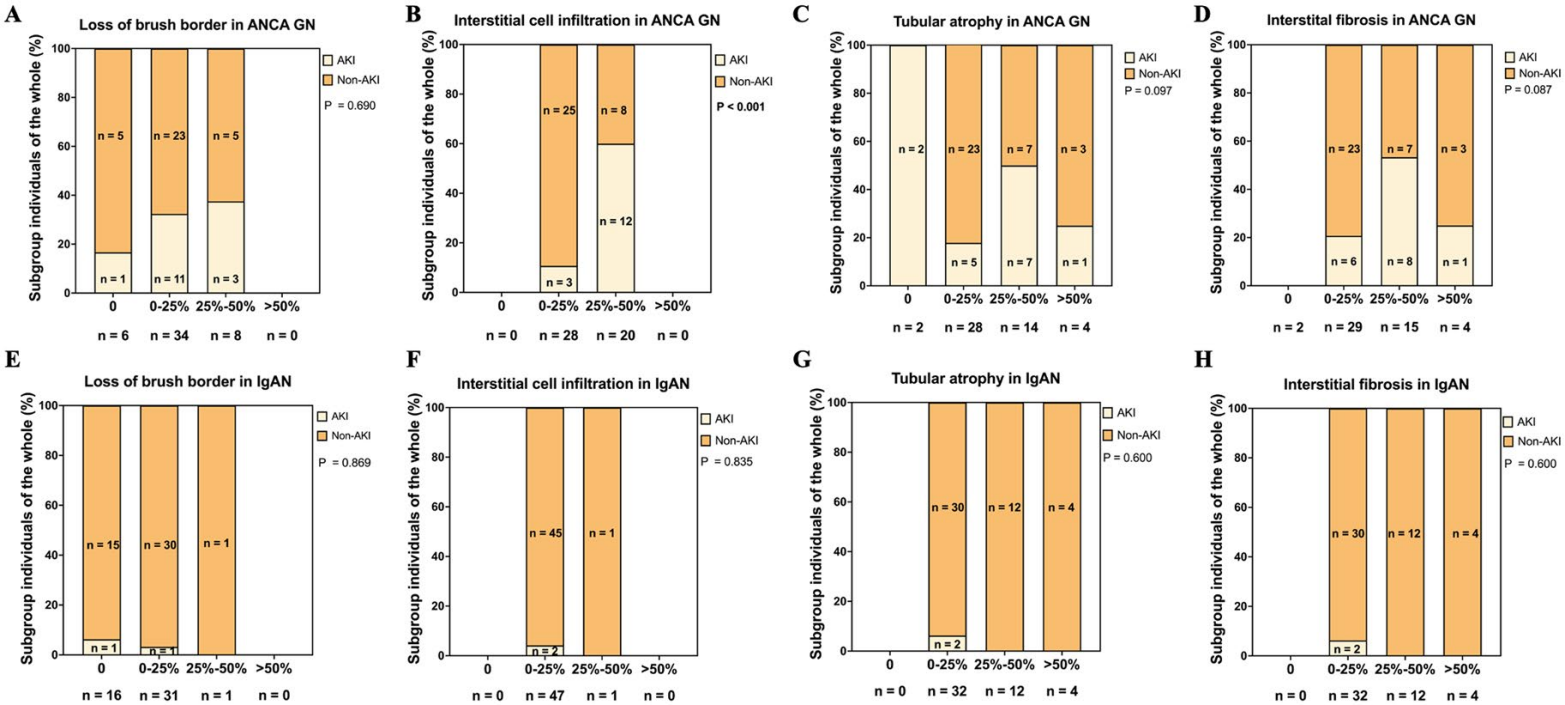
The association between AKI and tubulointerstitial injury in ANCA GN and IgAN. A The incidence of AKI in different level of loss of brush border in ANCA GN; B The incidence of AKI in different level of interstitial cell infiltration in ANCA GN; C The incidence of AKI in different level of tubular atrophy in ANCA GN; D The incidence of AKI in different level of interstitial fibrosis in ANCA GN; E The incidence of AKI in different level of loss of brush border in IgAN; F The incidence of AKI in different level of interstitial cell infiltration in IgAN; G The incidence of AKI in different level of tubular atrophy in IgAN; H The incidence of AKI in different level of interstitial fibrosis in IgAN.

As shown in Figure 2, among the 48 ANCA GN patients, 15 met the diagnostic criteria for AKI at the time of renal biopsy. Among the four parameters representing renal tubulointerstitial injury -loss of brush border, interstitial cell infiltration, tubular atrophy and interstitial fibrosis - the incidence of AKI was significantly higher in ANCA GN patients with interstitial cell infiltration >25% (*p* < 0.001), while the other three parameters were not associated with the incidence of AKI. Among the 48 IgAN patients, only two met the diagnostic criteria for AKI, and no parameter was found to be related to the occurrence of AKI in IgAN patients.

Next, we employed a logistic regression to identify which renal pathological parameters influenced the occurrence of AKI in patients with ANCA GN. In the univariate regression analysis, we found that both the glomerular crescent formation and the renal interstitial cell infiltration were risk factors for the development of AKI in ANCA GN patients. Subsequently, we performed a multivariate regression analysis with the crescent formation as a covariate. The results indicated that the renal interstitial cell infiltration remained an independent risk factor for AKI in ANCA GN (Table 3).

**Table 3.** Logistic regression of pathological risk factors for AKI in 48 ANCA GN patients.

Parameter	Univariate analysis			Multivariate analysis		
	OR	95% CI	*p* value	OR	95% CI	*p* value
Age	1.022	[0.981, 1.066]	0.297			
Sex, Female	1.300	[0.361, 4.679]	0.688			
Crescent fraction	480.638	[2.965, 77925.043]	**0.017**	1693.441	[3.284, 873154.275]	**0.020**
Fibrinoid necrosis fraction	0.007	[0.000, 583.958]	0.392			
Global glomerulosclerosis	13.559	[0.134, 1369.022]	0.268			
Bowman’s capsule rupture	1.538	[0.229, 10.326]	0.657			
Mesangial hypercellularity	0.905	[0.261, 3.134]	0.875			
Endocapillary hypercellularity	0.000	[0.000, -]	0.999			
Segmental glomerulosclerosis	1.107	[0.093, 13.248]	0.936			
Loss of brush border	1.593	[0.501, 5.063]	0.430			
Interstitial cell infiltration	12.500	[2.804, 55.731]	**<0.001**	16.426	[2.833, 95.241]	**0.002**
Tubular atrophy	1.157	[0.489, 2.738]	0.740			
Interstitial fibrosis	1.880	[0.742, 4.769]	0.183			

### Among ANCA GN patients with equivalent glomerular injury, those with higher interstitial cell infiltration also have higher serum creatinine levels

We conducted a comparison of the renal injury status among 48 ANCA GN patients who exhibited varying degrees of interstitial cell infiltration. As shown in Table 4, compared with 28 patients with interstitial cell infiltration of 0-25%, 20 patients with interstitial cell infiltration > 25% had higher serum creatinine levels. There were no significant differences in ANCA subtype, systematic disease activity, urine specific gravity, urine red blood cells, urine protein, urine NAG and urine NGAL levels between the two groups. To avoid the interference of glomerular injury on the results, we matched 16 patients with consistent levels of age, glomerular crescent formation, glomerular FN and glomerular global sclerosis in both groups for re-comparison. We found that when the influence of glomerular injury was excluded, patients with interstitial cell infiltration > 25% still had higher serum creatinine levels than patients with interstitial cell infiltration of 0-25% (147.00 [114.75, 239.50] vs 87.50 [68.75, 165.00] umol/L, *p* = 0.044). Regarding treatment, there were no significant differences in the use of RASI (12.50% vs. 6.25%, *p* = 0.544), glucocorticoids (81.25% vs. 75.00%, *p* = 0.669), immunosuppressive agents (31.25% vs. 43.75%, *p* = 0.465), or rituximab (31.25% vs. 43.75%, *p* = 0.465).

**Table 4.** Impact of interstitial cell infiltration on the severity of kidney injury in ANCA GN.

Parameter	Comparison without matching the severity of pathological indices of glomerular injury			Comparison after matching the severity of pathological indices of glomerular injury		
	Interstitial cell infiltration 0–25% (*N* = 28)	Interstitial cell infiltration >25% (*N* = 20)	*p* value	Interstitial cell infiltration 0–25% (*N* = 16)	Interstitial cell infiltration >25% (*N* = 16)	*p* value
Scr (μmol/L)	87.50 [63.50, 134.75]	141.50 [106.50, 212.50]	**0.003**	87.50 [68.75, 165.00]	147.00 [114.75, 239.50]	**0.044**
uSG	1.01 [1.01, 1.02]	1.01 [1.01, 1.02]	0.719	1.01 [1.01, 1.02]	1.01 [1.01, 1.02]	0.861
uRBC (/μL)	67.00 [18.33, 316.48]	205.30 [47.90, 375.00]	0.242	132.60 [27.58, 253.55]	183.85 [42.62, 307.42]	0.678
Proteinuria (g/24h)	1.09 [0.53, 2.83]	1.09 [0.71, 1.90]	0.809	1.38 [0.76, 2.91]	1.09 [0.70, 1.75]	0.419
uNAG (U/gcr)	22.30 [15.63, 29.80]	26.20 [13.20, 48.40]	0.418	23.15 [17.73, 28.03]	14.90 [13.20, 48.40]	0.95
uNGAL (ng/mL)	24.35 [15.34, 37.23]	42.90 [15.31, 64.84]	0.222	18.36 [14.90, 30.37]	43.50 [24.34, 72.75]	0.139
MPO-ANCA positive, n (%)	21 (75.00%)	19 (95.00%)	0.067	12 (75.00%)	15 (93.75%)	0.144
PR3-ANCA positive, n (%)	7 (25.00%)	1 (5.00%)	0.067	4 (25.00%)	1 (6.25%)	0.144
BVAS	8.50 [3.00, 21.75]	9.50 [2.25, 36.00]	0.638	8.50 [3.00,24.00]	8.50 [2.25, 36.00]	0.807
Treatment						
RASI Treatment, n (%)	2 (7.14%)	3 (15.00%)	0.380	1 (6.25%)	2 (12.50%)	0.544
Glucocorticoid treatment, n (%)	21 (75.00%)	17 (85.00%)	0.400	12 (75.00%)	13 (81.25%)	0.669
Immunosuppressive agents treatment, n (%)	11 (39.29%)	8 (40.00%)	0.960	7 (43.75%)	5 (31.25%)	0.465
RTX treatment, n (%)	11 (39.29%)	6 (30.00%)	0.507	7 (43.75%)	5 (31.25%)	0.465

**Abbreviations** Scr: Serum creatinine; uSG: Urinary specific gravity; uRBC: Urinary red blood cell; uNAG: Urinary N-acetyl-β-D-glucosaminidase; uNGAL: Urinary neutrophil gelatinase-associated lipocalin; RASI Renin-angiotensin system inhibitor treatment; RTX Rituximab treatment.

### Severe interstitial cell infiltration in ANCA GN patients is associated with high levels of peripheral blood leukocytes

To investigate the factors influencing the severity of renal interstitial cell infiltration in patients with ANCA GN, we performed a logistic regression analysis among all 48 ANCA GN patients. As presented in Table 5, in the univariate regression analysis, the high degree of renal interstitial cell infiltration (>25%) was associated with low level of hemoglobin (OR = 0.961, 95%CI [0.931, 0.992], *p* = 0.014), high level of peripheral blood leukocytes (OR = 1.423, 95%CI [1.122, 1.804], *p* = 0.004), and high level of serum creatinine (OR = 1.009, 95%CI [1.000, 1.018], *p* = 0.043). No association was found between crescent formation and interstitial cell infiltration (*p* = 0.323). In the multivariate regression analysis, only the level of peripheral blood leukocytes was identified as an independent risk factor for renal interstitial cell infiltration (OR = 1.449, 95%CI [1.111, 1.890], *p* = 0.006).

**Table 5.** Logistic regression of potential risk factors for high level of interstitial cell infiltration (>25%) in 48 ANCA GN patients.

Parameter	Univariate analysis			Multivariate analysis		
	OR	95% CI	*p* value	OR	95% CI	*p* value
Age	1.019	[0.982, 1.058]	0.311			
Sex, Female	1.202	[0.365, 3.956]	0.762			
BVAS	1.020	[0.991, 1.049]	0.186			
Hb (g/L)	0.961	[0.931, 0.992]	**0.014**	0.961	[0.922, 1.000]	0.052
WBC (*10^9^/L)	1.423	[1.122, 1.804]	**0.004**	1.449	[1.111, 1.890]	**0.006**
PLT (*10^9^/L)	1.004	[0.998, 1.010]	0.182			
Serum Alb (g/L)	0.923	[0.835, 1.020]	0.115			
D-Dimer (ng/mL)	1.000	[1.000, 1.001]	0.079			
ESR (mm/h)	1.025	[0.990, 1.061]	0.161			
CRP (mg/dL)	1.084	[0.963, 1.221]	0.181			
Serum C3 (mg/dL)	1.004	[0.972, 1.036]	0.830			
Serum C4 (mg/dL)	0.996	[0.919, 1.079]	0.919			
Scr (μmol/L)	1.009	[1.000, 1.018]	**0.043**	1.005	[0.995, 1.016]	0.330
uSG	0.000	[0.000, 0.000]	0.736			
uNAG (U/gcr)	1.038	[0.984, 1.096]	0.169			
uNGAL (ng/mL)	1.020	[0.994, 1.047]	0.132			
uRBC (/μL)	1.000	[1.000, 1.000]	0.635			
Proteinuria (g/24h)	0.804	[0.488, 1.326]	0.393			
Crescent fraction (%)	7.714	[0.134, 443.392]	0.323			
Fibrinoid necrosis fraction (%)	0.025	[0.000, 501.837]	0.465			
Global glomerulosclerosis (%)	1.677	[0.019, 148.439]	0.821			
Bowman’s capsule rupture	2.294	[0.346, 15.197]	0.389			

**Abbreviations** BVAS: Birmingham Vasculitis Activity Score; Hb: Hemoglobin; WBC: White blood cell; PLT: Platelet; Alb: Albumin; ESR: Erythrocyte sedimentation rate; CRP: C-reactive protein; C3: Complement 3; C4: Complement 4; Scr: Serum creatinine; uSG: Urinary specific gravity; uNAG: Urinary N-acetyl-β-D-glucosaminidase; uNGAL: Urinary neutrophil gelatinase-associated lipocalin; uRBC: Urinary red blood cell.

**Figure 3.**
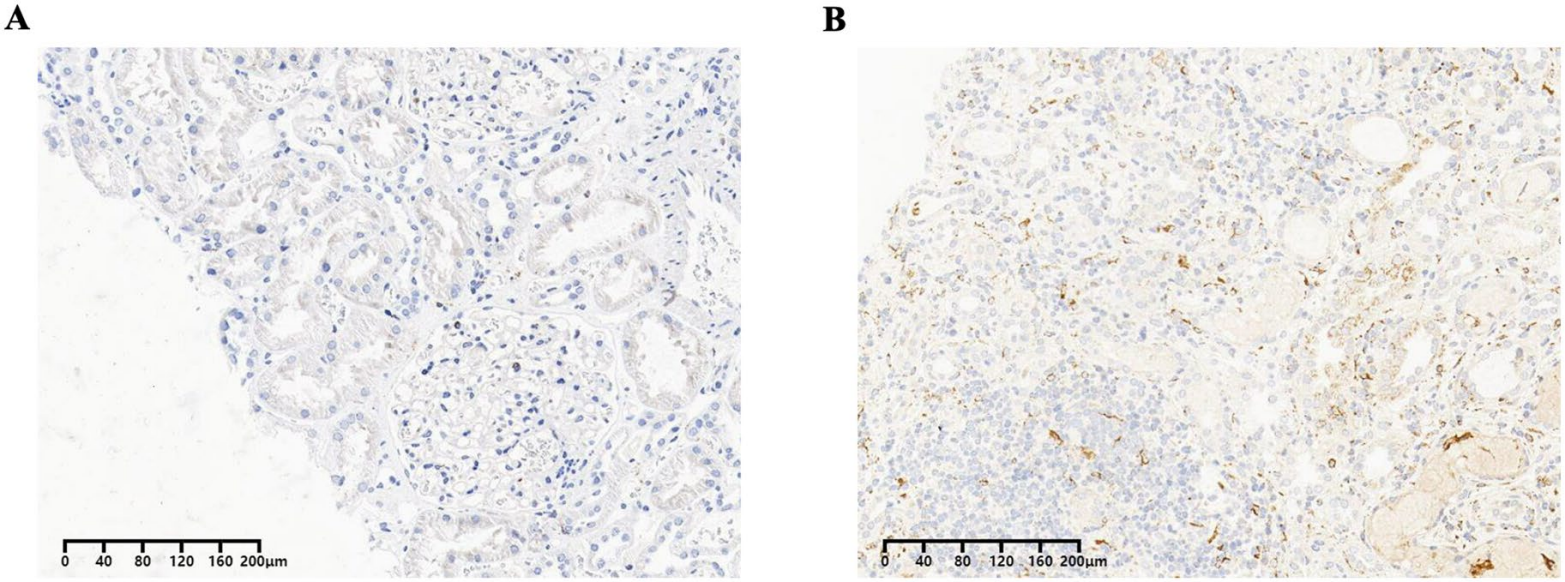
The staining for CD68 in ANCA GN kidney tissue. A Representative image showing minimal CD68-positive cell infiltration in an area with slight tubulointerstitial injury. B Representative image demonstrating dense infiltration of CD68-positive macrophages in an area of severe tubulointerstitial injury.

To phenotypically characterize the interstitial inflammatory infiltrate, we performed CD68 immunohistochemistry on selected cases. As demonstrated in Figure 3, renal tissue with severe tubulointerstitial injury exhibited a markedly greater density of CD68-positive macrophages within the expanded interstitium compared to tissue with only mild injury. This visual evidence corroborates the histopathological assessment of interstitial cell infiltration and indicates that macrophages constitute a significant component of the acute inflammatory infiltrate in ANCA GN.

### Interstitial cell infiltration has no effect on the renal prognosis of ANCA GN

**Figure 4.**
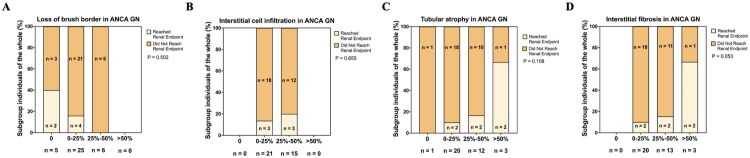
The association between renal outcome and tubulointerstitial injury in ANCA GN. A The incidence of renal endpoint in different level of loss of brush border in ANCA GN; B The incidence of renal endpoint in different level of interstitial cell infiltration in ANCA GN; C The incidence of renal endpoint in different level of tubular atrophy in ANCA GN; D The incidence of renal endpoint in different level of interstitial fibrosis in ANCA GN.

Among all 48 ANCA GN patients, a total of 36 patients had follow-up data and 6 patients reached the renal endpoint. As shown in Figure 4, none of the four parameters representing renal tubulointerstitial injury were found to be associated with renal prognosis. However, the degree of renal interstitial fibrosis exhibited a trend of positive correlation with renal endpoint events (*p* = 0.053). Since almost no IgAN patients reached the renal endpoint during the follow-up period, we did not analyze the relationship between tubulointerstitial injury and renal endpoint events in IgAN.

## Discussion

ANCA GN has been regarded as a glomerular disease for many years [15]. Although there have been some case reports of AAV interstitial nephritis, these cases were often considered as atypical ANCA GN [6,7,16–18]. ANCA GN is clinically classified as type III rapidly progressive glomerulonephritis. However, some phenomena have caught our attention. Some patients, although their serum creatinine levels rose rapidly at the onset of the disease, the proportion of glomeruli with crescent formation in their renal biopsies was not very high. In contrast, IgAN, one of the causes of type II rapidly progressive glomerulonephritis, frequently exhibits crescent formation on biopsy. However, a substantial proportion of these patients present clinically with only hematuria and proteinuria, without significant impairment of renal function, highlighting a spectrum of disease severity that differs markedly from the typically more aggressive acute presentation seen in ANCA-GN. Moreover, the serum creatinine levels of many ANCA GN patients dropped rapidly after glucocorticoid treatment, and this could not be solely attributed to ‘crescent absorption’. These phenomena suggest that glomerular injury is not the only factor determining the severity of AKI in ANCA GN. This comparative study between ANCA GN and IgAN provides compelling evidence that acute tubulointerstitial injury, particularly interstitial inflammatory cell infiltration, plays a critical and underappreciated role in the pathogenesis of AKI in patients with ANCA GN.

The principal finding of our study is that despite meticulous matching for the degree of glomerular injury—specifically, the proportions of cellular crescents, fibrinoid necrosis, and global glomerulosclerosis—ANCA GN patients exhibited significantly more severe acute tubulointerstitial damage, quantified by a greater loss of tubular brush border and a higher degree of interstitial cell infiltration, compared to their IgAN counterparts. This disparity in tubulointerstitial injury was paralleled by a more severe clinical presentation in ANCA GN patients, including higher serum creatinine levels and a markedly greater incidence of AKI, even though IgAN patients demonstrated more pronounced glomerular mesangial and endocapillary hypercellularity. This dissociation between glomerular and tubulointerstitial pathology strongly suggests that factors beyond the well-characterized glomerular crescentic and necrotizing lesions are major contributors to the acute loss of renal function in ANCA GN.

Furthermore, our analysis pinpointed interstitial cell infiltration as the key tubulointerstitial parameter independently associated with the development of AKI in ANCA GN. Multivariate regression analysis confirmed that interstitial inflammation remained a significant risk factor for AKI even after adjusting for the presence of glomerular crescents. This finding was reinforced by the sub-analysis within the ANCA GN cohort, which demonstrated that patients with more severe interstitial cell infiltration (>25%) had higher serum creatinine levels even after controlling for the extent of glomerular injury through additional matching. This underscores that the acute interstitial inflammatory process is a primary driver of functional impairment, potentially through mechanisms such as cytokine release [19–21], vascular compromise [22,23], and direct tubular toxicity [24], leading to reduced glomerular filtration and acute renal failure.

The exploration of potential drivers of this pronounced interstitial inflammation revealed a significant correlation with systemic inflammation. A high level of peripheral blood leukocytes was identified as an independent risk factor for severe renal interstitial cell infiltration. This suggests that the aggressive interstitial inflammation in ANCA GN may not be an isolated renal event but rather a local manifestation of the systemic inflammatory state characteristic of active AAV. Our immunohistochemical findings further substantiate this link by demonstrating that macrophages, regarding as CD68-positive cells, are a dominant component of the interstitial infiltrate in severe cases. Neutrophils, and monocytes activated by ANCA could directly infiltrate the interstitium, where monocytes likely differentiate into activated macrophages [25], perpetuating a cycle of injury and inflammation [25]. This provides a plausible cellular explanation for the observed acute functional impairment and highlights a potential therapeutic target beyond the initial ANCA-mediated neutrophil activation [26].

An intriguing and somewhat unexpected finding was that the severity of acute interstitial cell infiltration did not translate into a worse long-term renal prognosis during the follow-up period in our cohort. Instead, chronic changes like interstitial fibrosis showed a non-significant trend toward predicting endpoint events. This may be attributed to several factors. Firstly, the acute inflammatory component is often highly responsive to immunosuppressive therapy administered upon diagnosis [26–28]. Effective induction therapy can potentially halt and reverse acute inflammation, thereby mitigating its long-term impact. Secondly, our study’s follow-up duration might have been insufficient to capture the full progression of chronic damage initiated by acute episodes. Finally, it is the transition from acute inflammation to irreversible fibrosis that ultimately determines long-term outcomes [26,29–31]. The initial aggressive inflammation might set the stage for fibrogenesis, but the prognosis is likely dictated by the success of treatment in preventing this transition and the burden of chronic sclerotic lesions already present.

Our results illuminate a critical pathophysiological insight: acute tubulointerstitial inflammation is a major determinant of AKI in ANCA GN, distinct from and additive to the damage caused by glomerular lesions. This finding challenges the traditional glomerulocentric view of ANCA GN-related AKI. The strong association with peripheral leukocytosis further highlights the interplay between systemic and renal inflammation. From a clinical perspective, our findings argue for the incorporation of standardized assessment of tubulointerstitial inflammation in renal biopsy reports for ANCA GN, as it provides crucial prognostic information regarding the acute phase of the disease. Future therapeutic strategies might consider not only targeting the ANCA-mediated glomerular injury but also addressing the concomitant interstitial inflammatory response to more effectively preserve renal function in the acute setting.

To our knowledge our study was the first attempt to identify the potential mechanism leading to the acute kidney injury in ANCA GN by comparing with IgAN. However, limitations in the present study should be considered as well. One of the most limitations of this study was the relatively small number of included patients which might bias the statistical results. Future studies with larger, multicenter cohorts or those specifically designed to enroll matched AKI patients from both diseases will be valuable to validate and extend our findings regarding the differential role of tubulointerstitial inflammation in acute renal injury. Furthermore, the single center retrospective design makes it difficult to standardize treatments and explore whether the difference result was found in different racial people. As this study is susceptible to subtle differences in pathological findings, a prospective design rather than a retrospective one should be a more reasonable choice. Besides macrophage, we did not detail other types of inflammatory cell infiltration utilizing highly specific biomarkers, and the quantification of interstitial infiltration, while standardized, remained a semi-quantitative histological assessment. In addition, the comparison in this study is actual between two different kinds of diseases: one is an aggressive glomerulonephritis associated with a systemic disease, and the other is a relatively benign disease confined to the kidneys. Therefore, apart from interstitial renal injury, there may be some other potential factors influencing AKI that are difficult to rule out.

## Conclusion

Through a rigorously controlled design, we observed that the acute tubulointerstitial injury in ANCA GN was significantly more severe than that in IgAN, despite similar levels of crescent formation, fibrinoid necrosis and global glomerulosclerosis. The degree of interstitial cell infiltration exhibited a positive correlation with the number of peripheral blood leukocytes and had a substantial impact on AKI in ANCA GN. Consequently, the tubulointerstitial injury in ANCA GN cannot be regarded merely as secondary to glomerular injury; rather, its underlying mechanisms warrant further investigation.

## Data Availability

Raw data used during the current study are available from the corresponding author on reasonable request for noncommercial use.
